# Bet v 1-displaying elastin-like polypeptide nanoparticles induce a strong humoral and weak CD4+ T-cell response against Bet v 1 in a murine immunogenicity model

**DOI:** 10.3389/fimmu.2022.1006776

**Published:** 2022-10-06

**Authors:** Jolinde van Strien, Hans Warmenhoven, Adrian Logiantara, Max Makurat, Lorenz Aglas, Athanasios Bethanis, Romain Leboux, Leonie van Rijt, J. Andrew MacKay, Johannes W. van Schijndel, Gregory Schneider, René Olsthoorn, Wim Jiskoot, Ronald van Ree, Alexander Kros

**Affiliations:** ^1^ Department of Supramolecular and Biomaterials Chemistry, Leiden Institute of Chemistry, Leiden University, Leiden, Netherlands; ^2^ Department of Experimental Immunology, Amsterdam University Medical Centers, Amsterdam, Netherlands; ^3^ R&D Department, Haarlems Allergenen Laboratorium (HAL) Allergy B.V., Leiden, Netherlands; ^4^ Division of Allergy and Immunology, Department of Biosciences, Paris Lodron University of Salzburg, Salzburg, Austria; ^5^ Department of BioTherapeutics, Leiden Academic Centre for Drug Research (LACDR), Leiden University, Leiden, Netherlands; ^6^ Department of Pharmacology and Pharmaceutical Sciences, School of Pharmacy, University of Southern California, Los Angeles, CA, United States; ^7^ Department of Otorhinolaryngology, Amsterdam University Medical Centers, Amsterdam, Netherlands

**Keywords:** Bet v 1, nanoparticles, aluminum, hypo-allergenic, elastin-like polypeptide, mouse model

## Abstract

There is growing concern about the toxicity of colloidal aluminum salts used as adjuvants in subcutaneous allergen immunotherapy (SCIT). Therefore, alternative adjuvants and delivery systems are being explored to replace alum in SCIT. We applied micellar elastin-like polypeptides (ELPs), a type of self-assembling protein, to replace alum as vaccine adjuvant in birch pollen SCIT. ELP and an ELP-Bet v 1 fusion protein were expressed in *E. coli* and purified by immuno-affinity chromatography and inverse-transition cycling (ITC). Nanoparticles self-assembled from ELP and a 9:1 ELP/ELP-Bet v 1 mixture were characterized by using dynamic light scattering and atomic force microscopy. Allergenicity was assessed by measuring mediator release from rat basophilic leukemia cells transformed with the human FcϵR1 and sensitized with sera derived from human birch pollen allergic patients. Humoral and T-cell immunity were investigated by immunizing naïve mice with the ELP/ELP-Bet v 1 nanoparticles or alum-adsorbed Bet v 1, both containing 36 µg Bet v 1. ELP and ELP/ELP-Bet v 1 self-assembled at 37°C into spherically shaped micelles with a diameter of ~45 nm. ELP conjugation made Bet v 1 hypo-allergenic (10-fold). Compared to alum-adsorbed Bet v 1, ELP/ELP-Bet v 1 nanoparticles induced stronger IgG responses with an earlier onset. Additionally, ELP/ELP-Bet v 1 did not induce Th2 skewing cytokines and IgE. The hypoallergenic character and strong humoral immune response in the absence of a Th2-skewing T-cell response make ELP-based nanoparticles a promising candidate to replace alum in SCIT.

## Introduction

For more than 100 years, subcutaneous allergen immunotherapy (SCIT) using allergen extracts has been a disease-modifying allergy treatment (1). Despite its proven efficacy, therapy adherence is low due to the long treatment duration of SCIT, 3-5 years, and frequent occurrence of allergic side-effects. SCIT frequently contains colloidal aluminum salt-based adjuvants, such as aluminum hydroxide (alum) and aluminum phosphate. Allergens adsorbed to alum are partially protected from IgE binding, which reduces the risk of allergic side-effects (2). During SCIT, alum aids induction of mixed Th1/Th2 and regulatory T-cell and B-cell responses (3). Besides IL-10 production by the regulatory T- and B-cells, the regulatory B-cells also produce allergen-specific IgG_4_ antibodies that block IgE antibodies from binding to the allergen (4). In mouse models, allergen specific IgG can exert a similar blocking effect and IL-10 is associated with improved clinical outcome as well (5, 6).

Alum has been regarded as a safe adjuvant in vaccines for infectious diseases and SCIT for a long time (7). Nevertheless, there is growing concern about chronic alum exposure during SCIT, especially in a pediatric setting (8). Therefore, there is a growing demand to find alternatives for alum in SCIT (9). Recently, attention has focused on different types of nanoparticles to replace alum as allergy vaccine delivery system and adjuvant (10).

Nanoparticles are a promising delivery technology to improve the efficacy and safety of vaccines. Nanoparticle-based adjuvants can increase uptake and effective epitope presentation by antigen presenting cells and form a depot at the injection site which increases the duration of antigen exposure (11). Moreover, various nanoparticle types induce local release of cytokines, chemokines and damage-associated molecular patterns (DAMPs) resulting in immune cell recruitment and NLRP3 inflammasome activation, in turn stimulating a variety of downstream processes essential for adaptive immunity (11). Elastin-like polypeptide nanoparticles (ELPs) are based on artificial proteins modelled on human tropo-elastin (12, 13). ELPs comprise repeats of five amino acids (GVPGX)n in which X can be any amino acid and n determines molecular weight. ELPs undergo reversible hydrophobic collapse above the transition temperature (T_T_). In this study we used an amphiphilic ELP di-block copolymer consisting of two different repeating units that each have a different amino acid as the X residue. Above the critical micelle temperature (CMT), only the more hydrophobic block collapses, forcing the ELPs to self-assemble into micelles.

ELP-based assemblies are also of interest as drug carriers due to their inherent biodegradable, non-cytotoxic and non-immunogenic properties (12, 14–16). ELPs have therefore been used for various drug delivery applications (12) and are currently used in a clinical trial for pulmonary arterial hypertension (17). In addition, ELP micelles have been studied as vaccine carriers (18, 19). To our knowledge, ELPs have not been explored for use in SCIT until now.

ELP-based particles can be functionalized with a protein or peptide of interest by fusing both coding sequences and expressing the resulting fusion protein (20). The temperature dependent behavior of ELP conjugates is influenced by the hydrophobicity of the attached structure (20). The sequence of ELP conjugates can be designed to suit the purpose of the resulting particle. ELPs consisting of a single segment generally form so-called coacervates after injection (21–23). Such polypeptides can be used as a depot for treatment at the site of injection or for slow drug release (20, 24, 25). Alternatively, uniform micelles can be prepared with amphiphilic ELP conjugates. In this study we prepared such micelles, using a fusion protein of an amphiphilic ELP and Bet v 1 (**Table 1**), mixed with 9 equivalents of ELP (hereafter referred to as ELP/ELP-Bet v 1). We characterized these Bet v 1-displaying nanoparticles and compared them to alum-adsorbed Bet v 1 to investigate whether ELP based nanoparticles are a possible replacement for alum in SCIT.

**Table 1:** Sequences of proteins used in this study.

Name	amino acid sequence	MW (kDa)
ELP	M(GVPGI)_48_(GVPGS)_48_GY	39.6
ELP-Bet v 1	M(GVPGI)_48_(GVPGS)_48_-Bet v 1	56.8
Bet v 1	GVFNYETETTSVIPAARLFKAFILDGDNLFPKVAPQAISSVENIEGNGGPGTIKKISFPEGFPFKYVKDRVDEVDHTNFKYNY SVIEGGPIGDTLEKISNEIKIVATPDGGSILKISNKYHTKGDHEVKAEQVKASKEMGETLLRAVESYLLAHSDAYN	17.6

Proteins were expressed comprising the listed amino acid sequences. Both natural (no N-terminal methionine) and recombinant (expressed with an N-terminal methionine) Bet v 1 was used. No methionine was present between ELP and Bet v 1 in the ELP-Bet v 1 fusion protein.

## Experimental methods

### Chemicals and reagents

Pefablock was purchased from Roche Diagnostics; lysozyme was purchased from Thermo Scientific (Waltham, MA, US) and Benzonase from Sigma-Aldrich (St. Louis, MO, US). Recombinant Bet v 1 (Bet v 1.0101, hereafter called Bet v 1) was produced at the Department of Molecular Biology of the University of Salzburg, where it had been expressed in *E. coli* and purified according to previously established purification protocols (26). Cyanogen bromide-activated Sepharose 4B was purchased from GE Healthcare (Chicago, IL, US). Ultrapure water was obtained using a Milli-Q^®^ system. Birch pollen extract (BPE) containing 117 µg Bet v 1 per mg extract (as determined by ELISA) was obtained from HAL Allergy BV (Leiden, The Netherlands).

### Mice

Six to eight weeks old female BALB/c mice were purchased from ENVIGO (Venray, The Netherlands). The animals were housed under specific, pathogen free conditions at the animal facility of the Amsterdam UMC. All experiments were approved by the Animal Ethics Committee of the Amsterdam UMC.

### Construction of ELP-Bet v 1 plasmid

A pET25 expression vector coding for ELP was provided by the MacKay laboratory (27). The XbaI-AcuI (blunted by T4 DNA polymerase treatment) DNA fragment comprising the ELP sequence was recloned into XbaI-SmaI digested pET52b(+) and maintained in *E.coli* XL10-Gold. In the resulting pET52b-ELP plasmid XbaI and BseRI sites are available for inserting DNA fragments upstream, and Acc65I, BamHI, BsrGI, SalI, EagI, NotI, SacI, and AvrII sites are available for inserting DNA fragments downstream of the ELP coding sequence. pET52-ELP-Bet v 1 was constructed by cloning a synthetic DNA fragment of the Bet v 1 gene (BaseClear, Leiden, NL) into the Acc65I and NotI sites of pET52b-ELP.

### Expression of ELP and ELP-Bet v 1

ELP (M(GVPGI)_48_(GVPGS)_48_GY) and ELP-Bet v 1 (M(GVPGI)_48_(GVPGS)_48_-Bet v 1.0101) were expressed by transforming the plasmids into *E. coli* BL21(DE3) cells using the heat shock and calcium methods. The cell cultures were grown in LB medium containing ampicillin (250 μg/mL) at 37°C. Starter cultures of 10 mL were added to 1 L of medium and cultured until the OD600 was ~0.5. The cultures were cooled to 18°C and were induced overnight with 0.05 mM IPTG. The cells were harvested and washed with 0.9% NaCl solution. The cell pellets were frozen at -80°C. The cells were lysed in 10 mM phosphate buffer (PB) pH 7.8 containing 1 mM pefablock, 1 mg/mL lysozyme, 2 mM MgCl_2_, 25 u/mL benzonase in a total volume of 10 mL. The mixtures were incubated at 4°C for approximately 45 minutes and sonicated on ice at 25% amplitude for 5 minutes in 5 second intervals. The solids were removed from the lysates by centrifugation at 4°C and 37000 rpm (228783 rcf) for 30 minutes.

### Purification of ELP

ELP was purified by inverse transition cycling. NaCl was added to the lysate to reach a concentration of 4 M. Salt lowers the TT of ELPs, which enables aggregation of the polypeptide at room temperature for purification purposes. After incubation for 30 minutes at room temperature the mixture was centrifuged at 22°C and 10000 rpm (17100 rcf) for 30 minutes. The pellet was suspended in cold 10 mM PB (pH 7.8) and incubated for at least 30 minutes at 4°C. After centrifugation at 4°C and 10000 rpm (17100 rcf) for 30 minutes the dissolved ELP was collected by decanting the supernatant. The cycle of room temperature and 4°C incubation and centrifugation steps were repeated four times by using 3 M NaCl for the incubation at room temperature. The final supernatant was dialyzed against 10 mM PB pH 7.8.

### Purification of ELP-Bet v 1

The lysate was first purified by using immunoaffinity chromatography at 4°C. A Bet v 1 specific monoclonal antibody, 5H8H9 (28), was coupled to cyanogen bromide-activated Sepharose 4B according to manufacturer’s instructions. The lysate was loaded on the column that had been equilibrated with 10 mM PB. The column was washed with 5 column volumes of PB and eluted with 100 mM glycine pH 2.5. The fractions were immediately neutralized with 1 M Tris pH 8.8. The elution fractions containing ELP-Bet v 1 were combined and further purified by one cycle of inverse transition cycling as described above for the purification of ELP. The final ELP-Bet v 1 solution was dialyzed against 10 mM PB pH 7.8.

### SDS-PAGE

Samples for SDS-PAGE were mixed with reducing Laemmli buffer and loaded directly on a 10% SDS polyacrylamide gel. Electrophoreses was performed at 200 V. The gels with ELP-containing samples were washed with water for 5 minutes, stained with 0.5 M CuCl_2_ for 15 minutes and washed with water for 3 x 5 minutes. The gels used to analyze samples with ELP-Bet v 1 were stained with Coomassie Brilliant Blue R-250 (Bio-Rad, Hercules, CA, US).

### UV-VIS spectroscopy

The polypeptide concentrations were determined based on the absorbance at 280 nm. The absorbance was measured on a Cary 300 device at 10°C. The theoretical extinction coefficients for ELP (1490 M cm^-1^), ELP-Bet v 1 (10430 M cm^-1^) and Bet v 1 (10430 M cm^-1^) were calculated by the ProtParam tool of ExPasy.

### Dynamic light scattering and static light scattering

All DLS and SLS measurements were done on a Malvern Zetasizer Nano-S instrument. The cuvette containing the sample was placed in the cell that had been heated to 37°C or, in case of the CMT determination measurements, to the relevant temperature. ELP concentrations were 10 µM for the CMT determination, and 100 µM diluted to 1 µM for the instant dilution experiment. The measurement was started after 2 minutes of incubation time. For the CMC measurements, the attenuator was fixed at 11 to allow a direct comparison of the static light scattering intensities (count rates), whereas it was set to automatic for all other measurements.

### Zeta potential

Zeta potential measurements were performed on a Malvern Zetasizer Nano-ZS by using a Malvern Zetasizer nano series Universal Dip Cell kit. 1 mL of a 2.5 µM polypeptide solution in 10 mM PB was incubated at 37°C before starting the measurements. The results are averages of three runs, each comprising 12 measurements of 5 seconds.

### AFM

Samples for AFM were prepared by drop-casting 20 µL of 37°C 2 µM ELP or ELP/ELP-Bet v 1 on a silicon oxide wafer (Siegert Wafer) with a 285 nm thermal oxide layer or on a mica disc (V1 grade; Muscovite). The samples were dried at 37°C for 30 minutes. AFM images were recorded using a JPK NanoWizard Ultra Speed microscope and the obtained data was processed using the JPK SPM Data Processing software. All experiments were performed using a silicon probe (Olympus, OMCL-AC160TS) with a nominal resonance frequency of 300 kHz. The images were all scanned and recorded (with a resolution of 512x512 pixels) in intermittent contact mode in air at room temperature.

### IgE binding of ELP-Bet v 1

IgE binding of ELP-Bet v 1 and ELP/ELP-Bet v 1 was determined by ImmunoCAP™ IgE inhibition assay. The samples were diluted in 10 mM PB, 280 mM sucrose, pH 7.4. The concentrations of ELP-Bet v 1 and Bet v 1 were determined by UV-VIS spectrometry. A pool of 36 birch pollen allergic patient sera was diluted to 12 kU/mL IgE and added 1:1 (v/v) to the samples followed by incubation at room temperature for one hour. Uncomplexed IgE was measured on a Phadia™ 250 (Thermo-Scientific) with rBet v 1 ImmunoCAPs (t215), following the manufacturer’s instructions.

### RBL assay

The assay was performed by using a rat basophil (RBL-2H3) cell line, transfected with the human high-affinity IgE receptor (FcϵRI), as previously reported (26, 29). In short, 1 x 10^5^ RBL-2H3 cells per well were seeded in flat-bottom 96-well, Nunclon Delta-treated microplates (Thermo Fisher Scientific, Waltham, MA, USA) and passively sensitized with human sera derived from birch pollen allergic patients (n=10) in a final dilution of either 1:10 or 1:20. Before the sensitization step, sera were incubated with P3X63Ag8.653 cells (ATCC CRL-1580™, Manassas, VA, USA) to neutralize the complement system. To trigger the β-hexosaminidase release, the cells were stimulated for 1 hour at 37°C, 7% CO_2_, with the respective antigen in concentrations ranging from 80 µg/mL to 0.024 fg/mL (based on UV-VIS). The antigen concentration was based on the Bet v 1 concentration. As an additional control we prepared a 10:1 mix consisting of 10 parts ELP particles and 1 part Bet v 1. To detect β-hexosaminidase activity, the fluorogenic substrate, 4-methyl umbelliferyl-N-acetyl-beta-D-glucosaminide (Sigma-Aldrich) was used and measured at an excitation and emission wavelength of 360 nm and 465 nm, respectively. The data were corrected for spontaneous release (untreated cells) and normalized to the maximal enzyme release caused by cell lysis (10% Triton X-100, Sigma-Aldrich).

### Immunogenicity of ELP-Bet v 1 nanoparticles

For the first *in vivo* immunogenicity experiment, mice were immunized subcutaneously at days 0, 7 and 14 with 200 µL ELP-Bet v 1 and ELP/ELP-Bet v 1 containing 36 µg Bet v 1 (n=5-6) and 102 µM total polypeptide concentration or the equivalent amount of Bet v 1 adsorbed to alum. The Bet v 1 amount is based on the equivalent dose of Bet v 1 in BPE (300 µg BPE containing 36 µg Bet v 1) used in a birch pollen allergy SCIT mouse model (5). The ELP-Bet v 1 and ELP/ELP-Bet v 1 formulations were equilibrated to room temperature (~20°C) before injection. The antigen concentration was determined by UV-VIS spectrometry. ELP in phosphate buffered sucrose (10 mM PB pH 7.8, 280 mM sucrose) was used as negative control group (n=5). In the second experiment, the ELP-Bet v 1 group was replaced by a phosphate buffered sucrose alum group as negative control. The formulations contained low LPS levels (**Figure S1**). Serum immunoglobulin levels were measured in serum samples taken *via* puncture of the vena saphena at days -1, 6, 13 and 20. At day 28, 29 and 30 the animals received 100 µg BPE (containing 12 µg Bet v 1) in 30 µL PBS intranasally under 3% (v/v) isoflurane anesthesia to further boost antibody production. After sacrificing the mice on day 31, blood and lung draining lymph nodes were collected to analyze Bet v 1 specific IgG_1_, IgG_2a_, and IgE levels, and the production of IL-4, IL-5, IL-13, IL-10, IL-17A and IFN-γ, respectively.

### Analysis of serum Bet v 1 specific immunoglobulin levels

Bet v 1 specific IgE, IgG_1_ and IgG_2a_ antibodies in serum, collected at the different time points, were analyzed as described previously (30). Briefly, NUNC Maxisorp plates were coated overnight with 5 µg Bet v 1. The next day, the plates were blocked with FCS, followed by incubation with the serum samples. After washing, bound immunoglobulins were detected with horse radish peroxidase-conjugated specific antibodies against mouse IgE, IgG_1_ (Opteia, BD, San Diego, CA, USA) and IgG_2a_ (eBioscience), according to the manufacturer’s instructions. Serum samples of all groups were diluted 10 times for IgE detection. Serum samples for IgG_1_ and IgG_2a_ detection were diluted 100 times except for the ELP/ELP-Bet v 1 and alum-adsorbed Bet v 1 groups which were diluted between 100 and 10.000 times depending on the measured time point.

### *Ex vivo* re-stimulation of lung draining lymph node cells.

Lung draining lymph node cell suspensions were plated in 96 well round bottom plates (Sigma-Aldrich) at a density of 2 x 10^5^ cells per well and were re-stimulated for 4 days with Bet v 1. IL-4, IL-5, IL-10, IL-13, IFN-γ and IL-17A expression levels were determined in the supernatant by ELISA (eBioscience).

### Statistics

For the RBL test, the data was normalized based on the minimum and maximum values of the Bet v 1 series for each patient. For calculation of the antigen concentration necessary for half maximal release, the average of the maximal (normalized for maximal release) and minimal values (corrected for spontaneous release) of each curve were used. A repeated-measures one-way ANOVA followed by Tukey’s *post-hoc* analysis test was performed to determine significant differences among the treatment groups. Normal distribution was confirmed *via* QQ plot. IgG_1_ and IgG_2a_ levels were first log10 transformed and then analyzed with two-way ANOVA followed by Tukey’s multiple comparison test. IgE and cytokine levels were log10 transformed and subsequently analyzed with a one-way ANOVA followed by Tukey’s multiple comparison test. P-values <0.05 were considered significant.

## Results

### Purification of ELP and ELP-Bet v 1

**Figure 1:**
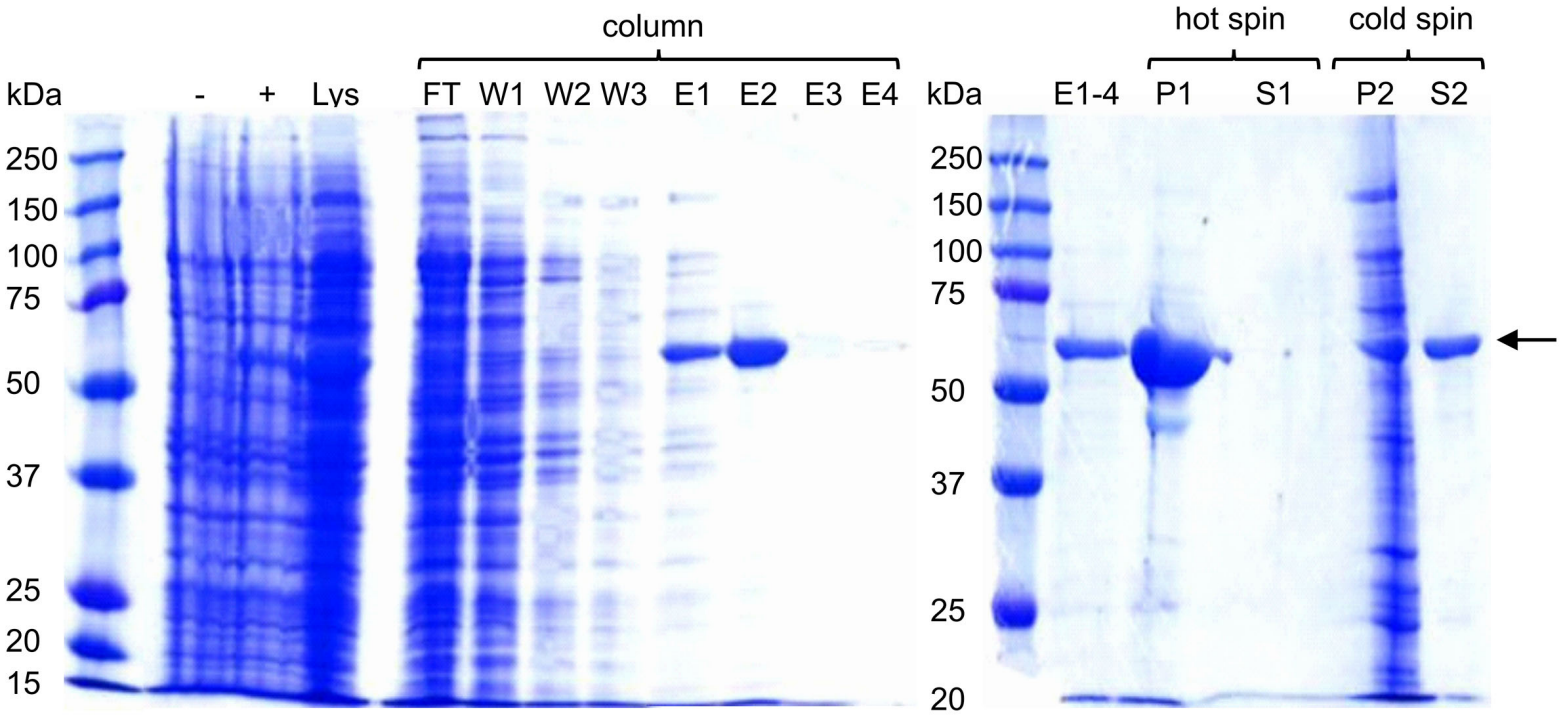
Expression and purification of ELP-Bet v 1. Samples were analyzed on a 10% SDS-PAGE gel using Coomassie Blue staining. ELP-Bet v 1 is clearly visible at about 57 kDa and is indicated with a black arrow. - and + refer to E. coli samples taken before and after induction with IPTG, respectively. Lys is lysate; FT is flowthrough; W is wash; E is elution; P is pellet; S is supernatant; hot spin is the centrifugation step at 22°C; cold spin is the centrifugation step at 4°C.

ELP was purified as previously described by Janib et al. (27) (**Figure S2**). ELP-Bet v 1 was first purified by immuno-affinity chromatography and then by a single cycle of inverse transition cycling (ITC) (**Figure 1**). The lysate containing ELP-Bet v 1 was loaded on a Bet v 1 specific monoclonal antibody column. Unbound proteins were collected (FT) and the column was washed (**Figure 1**, W1-3). ELP-Bet v 1 was eluted at pH 2.5 (E1-4). The combined elution fractions still contained some impurities. NaCl was added (3 M) to precipitate the polypeptide (P1), which was collected by centrifugation at room temperature. The supernatant did not contain any substantial amount of protein (S1). The pellet was resuspended in cold, 10 mM potassium phosphate (PB) and centrifuged again at 4°C. The protein impurities remained in the pellet (P2) while the supernatant (S2) contained purified ELP-Bet v 1. The exact mass of both ELP and ELP-Bet v 1 was confirmed by mass spectrometry (**Figure S3**) and the purity by analytical RP-HPLC (**Figure S4**).

### Physicochemical characterization of ELP-Bet v 1-based micelles

**Figure 2:**
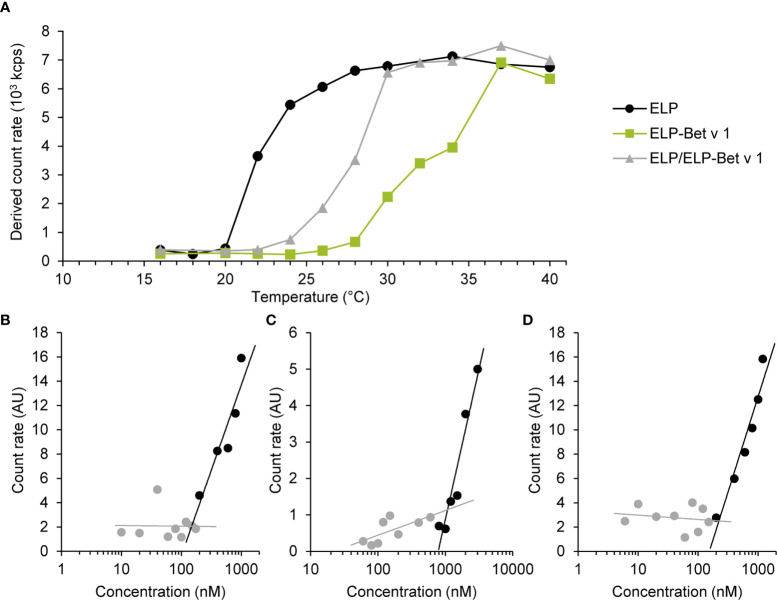
Critical micelle temperature **(A)** and concentration **(B–D)** of ELP, ELP-Bet v 1 and ELP/ELP-Bet v 1. **(A)** Polypeptide solutions of 10 µM in 10 mM PB were measured by SLS at varying temperatures. The CMT is determined as the temperature value above which the count rate exceeds the baseline measurement: ~22°C (ELP), 24°C (ELP/ELP-Bet v 1) and ~28°C (ELP-Bet v 1). **(B–D)** Various concentrations of ELP **(B)**, ELP-Bet v 1 **(C)** and ELP/ELP-Bet v 1 in 10 mM PB were measured with SLS at 37°C by using a fixed attenuator of 11. Count rates were normalized to the count rate of the buffer. Gray data represent samples that did not contain particles according to the autocorrelation functions (**Figure S5**); black data represent the samples for which the autocorrelation functions had a sigmoidal shape. CMCs at 37°C were determined by calculating the concentration at the intercept of both trend lines: 0.15 µM (ELP), 1.1 µM (ELP-Bet v 1) and 0.22 µM (ELP/ELP-Bet v 1).

**Figure 3:**
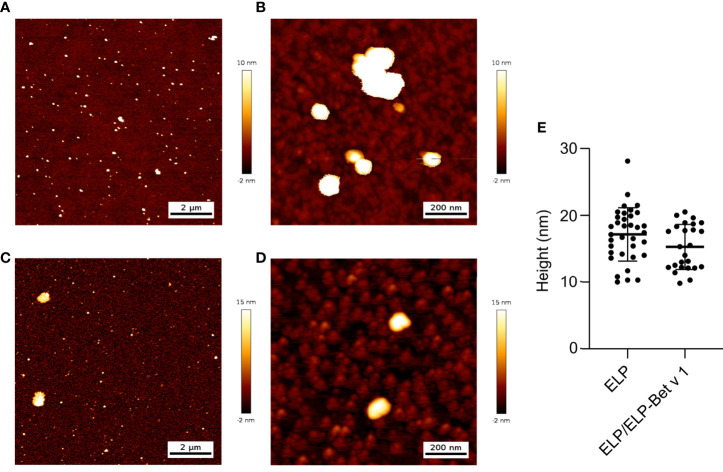
AFM images and analysis of ELP **(A, B)** and ELP/ELP-Bet v 1 **(C, D)**. Polypeptide solutions in water were heated to 37°C and deposited on a silicon oxide surface. The samples were dried at 37°C and imaged using height trace mode at medium **(A**, **C)**, high **(B, D)** and low (**Figure S6**) magnifications. The medium magnification was used to analyze the average height of the imaged micelles**(E)**: 17.1 nm (ELP) and 15.3 nm (ELP/ELP-Bet v 1). Error trace versions of these images are depicted in **Figure S6**. Control images and images of the samples on mica are compiled in **Figure S7**.

**Figure 4:**
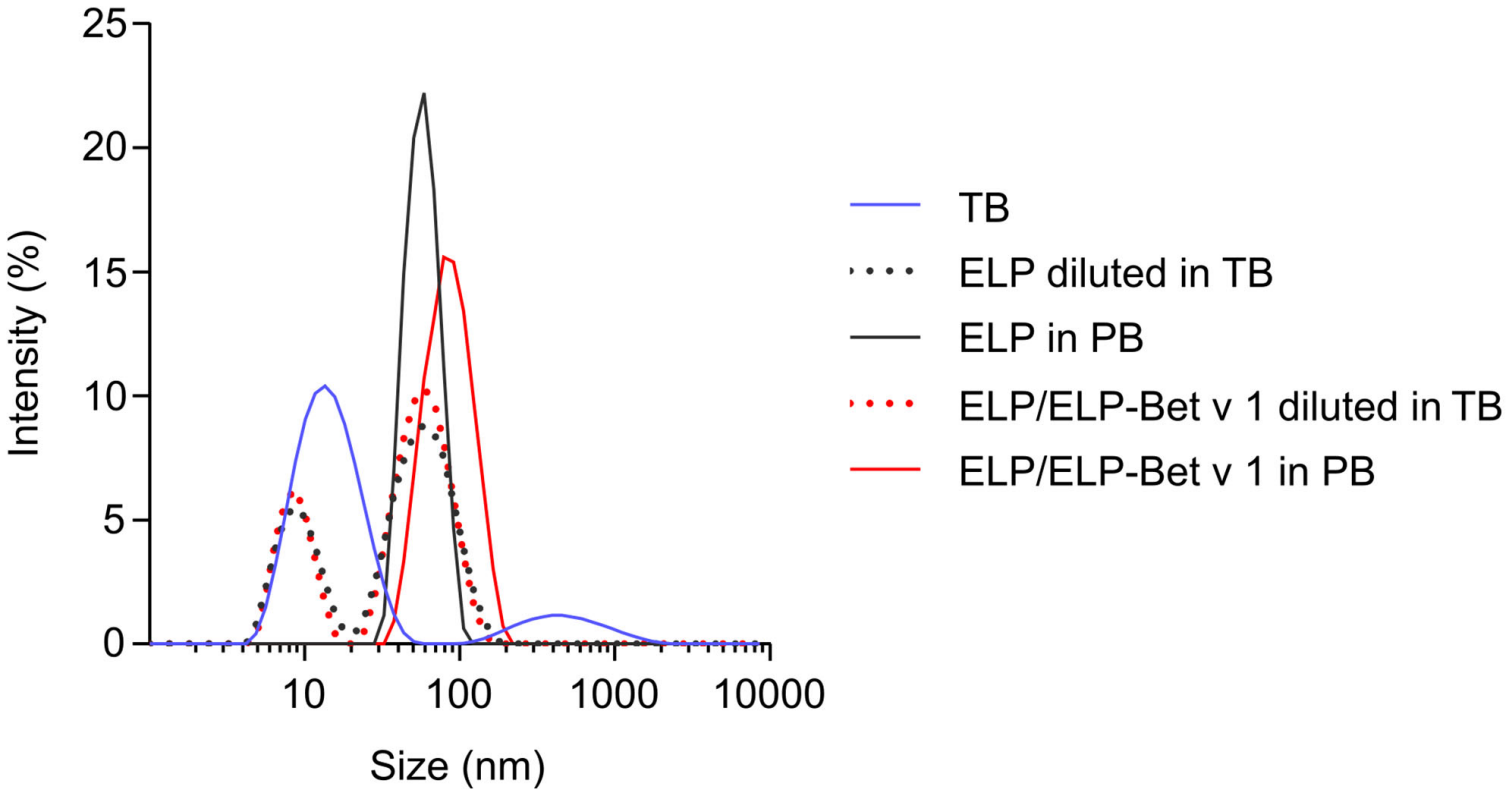
Rapid micellization of cold ELP/ELP-Bet v 1 in 37°C Tyrode’s buffer. DLS size distributions of either 1 µM polypeptide solution in 10 mM PB at 37°C or instantly diluted from 100 µM cold solution into 37°C Tyrode’s buffer (TB) to reach a final polypeptide concentration of 1 µM.

For the design of the birch pollen vaccine candidate we used ELPs with a CMT of approximately 22°C (27). ELP-Bet v 1 had a higher CMT of approximately 28°C and the CMT of ELP/ELP-Bet v 1 was 24°C (**Figure 2A**). The fusion of Bet v 1 to ELP also increased the critical micelle concentration (CMC) from 0.15 µM for ELP to approximately 1 µM for ELP-Bet v 1 (**Figures 2B, C**). The ELP/ELP-Bet v 1 that was used in this study had a CMC of 0.22 µM (**Figure 2D**). The higher CMC and CMT of ELP-Bet v 1 with respect to ELP/ELP-Bet v 1 illustrates the higher suitability for *in vivo* use of the mixture and was therefore selected as our primary vaccine candidate. AFM showed both ELP and ELP/ELP-Bet v 1 formed monodisperse micelles at 37°C (**Figure 3**, **Figures S6, S7**). The hydrodynamic diameter (Dh) of 44.6 nm as measured with DLS was larger than the 15 nm spherical nanoparticles as observed with AFM imaging (**Table 2**). This difference is to be expected, since hydrodynamic diameters of hydrated polymers are usually larger than imaged diameters of dried nanoparticles (31). Also, DLS is biased towards species with the largest diameter. Both ELP and ELP/ELP-Bet v 1 instantly formed micelles when 100 µM cold solution was diluted 100-fold in Tyrode’s buffer equilibrated at 37°C (**Figure 4**). This suggests micelles will form when a cold sample is injected into mice. The zeta potential of ELP particles and ELP/ELP-Bet v 1 particles was close to zero (**Table 2**), which is in congruence with the neutral charge of ELP. As expected, based on the negative charge of Bet v 1, ELP-Bet v 1 particles have a negative zeta potential (-12 mV).

**Table 2:** Size and zeta potential of ELP, ELP-Bet v 1 and ELP/ELP-Bet v 1.

Formulation	Dh (nm)	PdI	Ζeta potential (mV)
ELP	46.7 ± 0.2	0.087 ± 0.023	-4.3 ± 0.29
ELP-Bet v 1	50.8 ± 0.9	0.051 ± 0.007	-12.0 ± 1.8
ELP/ELP-Bet v 1	44.6 ± 0.3	0.045 ± 0.024	-6.8 ± 0.26

Polypeptide solutions of 10 µM in phosphate buffered sucrose (DLS) or 2.5 µM in 10 mM PB (zeta potential) were measured at 37°C. The corresponding size distributions are shown in **Figure S8**.

### ELP conjugation modulates IgE associated mediator release of Bet v 1

**Figure 5:**
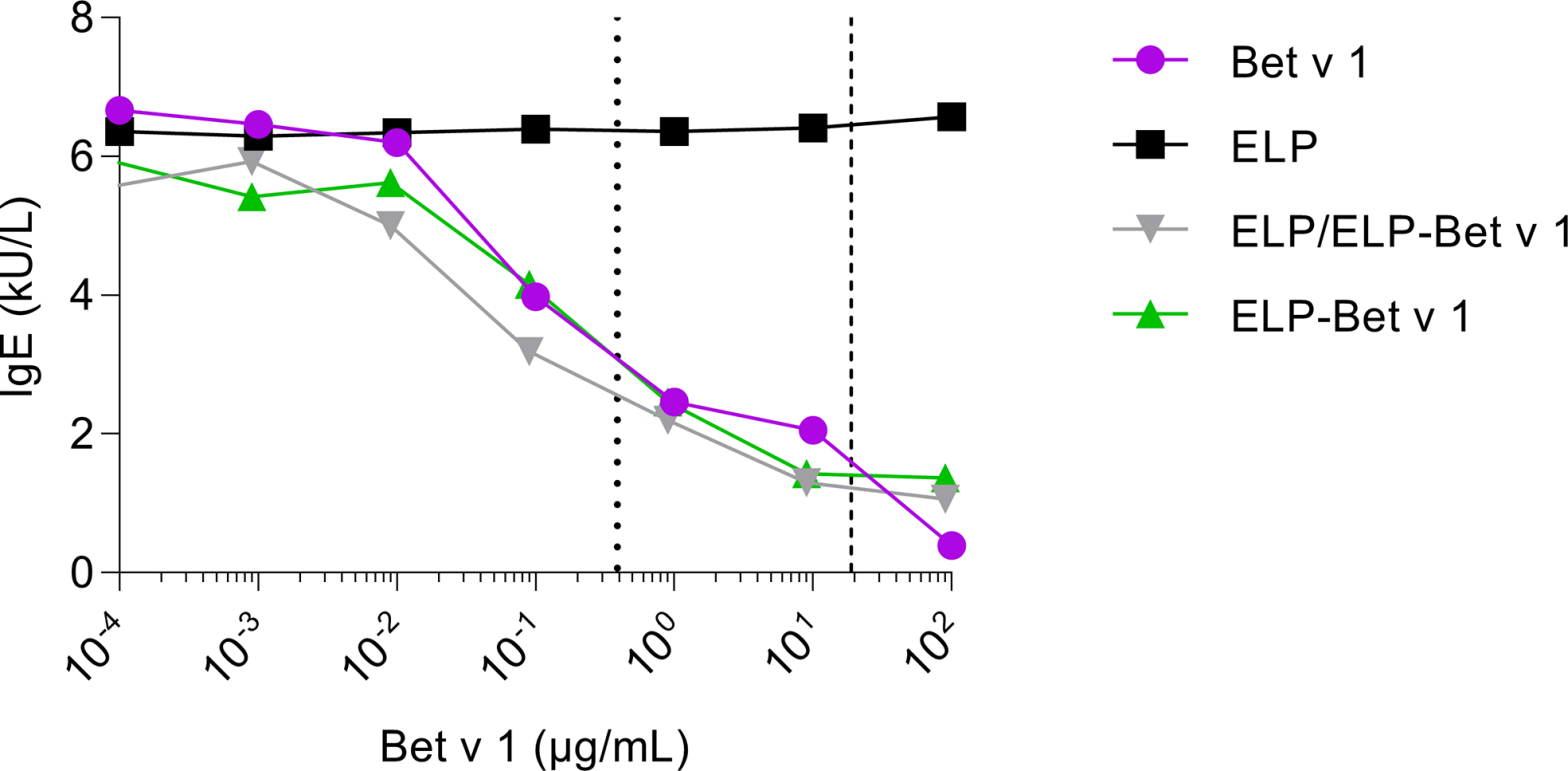
Inhibition of IgE binding to immobilized Bet v 1. ELP, Bet v 1, ELP-Bet v 1 and ELP/ELP-Bet v 1 were incubated at various concentrations with serum from birch pollen allergic patients containing Bet v 1-specific IgE. The mixtures were subsequently applied to rBet v 1 ImmunoCAPs containing immobilized Bet v 1. Next, the IgE levels in the eluted solutions were measured. The dashed and dotted lines indicate the CMC of ELP/ELP-Bet v 1 (0.39 µg/mL) and ELP-Bet v 1 (19 µg/mL), respectively.

**Figure 6:**
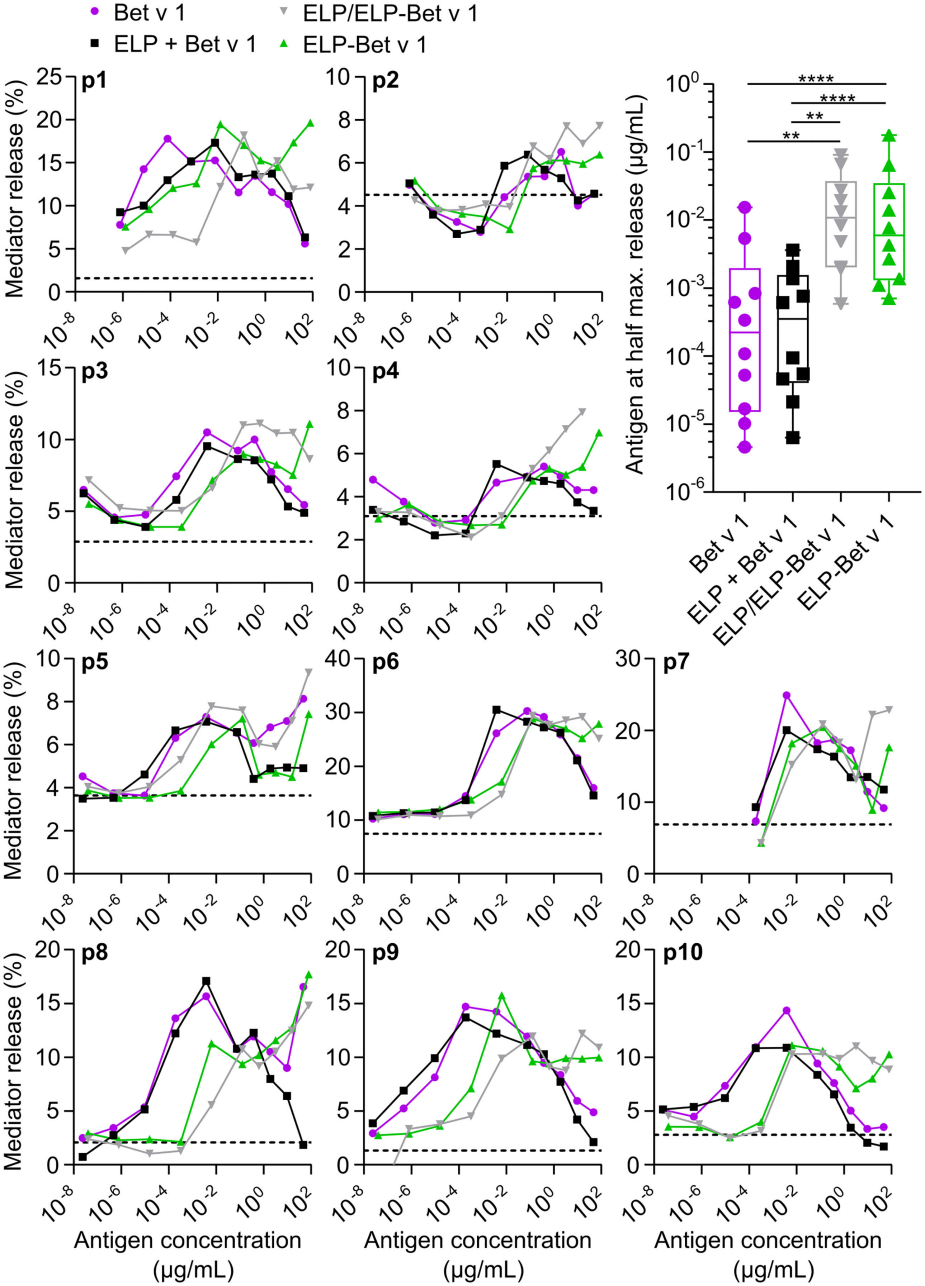
RBL assay. Mediator release from rat basophils displaying human IgE from 10 patients (p1-p10) were measured following incubation with a series of antigen concentrations. The level of mediator release in the absence of antigen is depicted as a dashed line. The CMC of ELP/ELP-Bet v 1 (0.39 µg/mL) and ELP-Bet v 1 (19 µg/mL) are located closely together but are not indicated in this figure to maintain clarity. Right shifted curves indicate higher concentrations required to get the same level of mediator release. This hypo-allergenicity is quantified by determining the Bet v 1 concentration at half the maximum value of mediator release on the left side of the peak. *p<0.05, **p<0.01, ***p<0.001, ****p<0.0001.

To determine to what extent Bet v 1 in ELP/ELP-Bet v 1 nanoparticles is recognized by IgE, we compared IgE binding capacity of the nanoparticles with that of soluble Bet v 1 by performing an IgE ImmunoCAP inhibition assay. The CMC of ELP-Bet v 1 and ELP/ELP-Bet v 1 correspond to 19 and 0.39 µg/mL Bet v 1, respectively. The observed similar Bet v 1, ELP-Bet v 1 and ELP/ELP-Bet v 1 IgE binding capacities indicate that IgE binding to Bet v 1 was not affected by ELP conjugation (below CMC) or micelle formation (above CMC). Moreover, the Bet v 1 moiety in ELP-Bet v 1 was correctly folded and still recognized by IgE (**Figure 5**). The correct folding of Bet v 1 is supported by circular dichroism (CD) spectra (**Figure S9**). Above the CMC, ELP-Bet v 1 and ELP/ELP-Bet v 1 seemed to induce increased mediator release with respect to Bet v 1 and plain ELP mixed with Bet v 1 (denoted “ELP + Bet v 1”) (**Figure 6**). However, below the CMC conjugation to ELP caused a right shift of the mediator release curve, meaning more Bet v 1 is needed to induce the same level of mediator release, as illustrated by half the maximum β-hexosaminidase release (**Figure 6**). This concentration was on average a 10-fold higher for ELP/ELP-Bet v 1 compared to free Bet v 1.

### ELP particles induce strong humoral and weak T-cell responses in naïve mice

**Figure 7:**
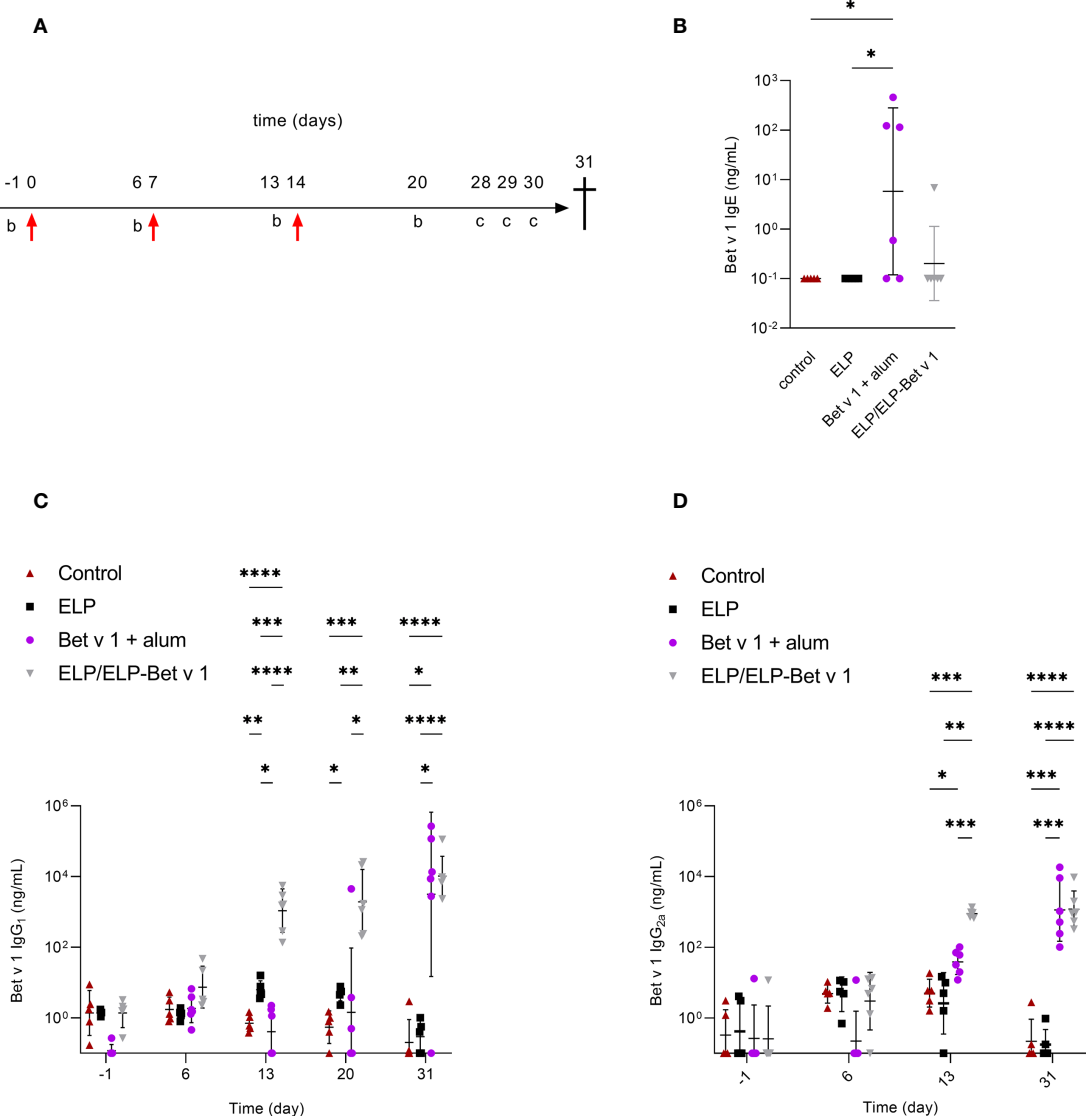
*In vivo* immunogenicity in naïve mice. **(A)** Immunization protocol. Mice were immunized with 36 µg Bet v 1 on day 0 by subcutaneous injection followed by booster immunizations on days 7 and 14 (red arrows). Blood samples were taken for serum immunoglobulin analyses on days -1, 6, 13 and 20. The mice received intranasal BPE challenges **(C)** to further boost immunoglobulin production on days 28, 29 and 30. At day 31 the mice were sacrificed. **(B)** Serum Bet v 1-specific IgE levels at day 31. **(C)** Serum Bet v 1-specific IgG_1_ levels on days -1, 6, 13, 20 and 31. **(D)** Serum Bet v 1-specific IgG_2a_ levels on days - 1, 6, 13 and 32. Day 20 data are not available because of incorrect storage of the corresponding serum samples. *p<0.05, **p<0.01, ***p<0.001 and ****p<0.0001.

In a pilot immunogenicity experiment in naïve mice, we investigated the immune responses of ELP/ELP-Bet v 1, alum-adsorbed Bet v 1, ELP-Bet v 1 and ELP (**Figure 7A**). ELP/ELP-Bet v 1 micelles induced significant IgG_2a_ levels at day 13 and at day 20, both IgG_1_ and IgG_2a_ levels in this group were significant (**Figures S10A, B**). In contrast, ELP, ELP-Bet v 1 and alum-adsorbed Bet v 1 treated mice did not exceed the endpoint IgG_1_ and IgG_2a_ levels typically observed in buffer only control groups from immunogenicity experiments performed in our lab. Compared to the ELP negative control group ELP/ELP-Bet v 1, ELP-Bet v 1 and alum-adsorbed Bet v 1 did not induce significant IgE levels at the endpoint (**Figure S10C**).

Subsequently, we further analyzed the immune response against the ELP/ELP-Bet v 1 nanoparticles upon subcutaneous administration in mice. As expected, alum-adsorbed Bet v 1 showed significant expression of Th2 cytokines IL-4, IL-5 and IL-13 (**Figure S11**). Furthermore, alum-adsorbed Bet v 1 showed expression of Treg related IL-10 but not Th1 cytokine IFN-γ and Th17-related cytokine IL-17A (**Figure S11**). Compared to ELP, both ELP-Bet v 1 and ELP/ELP-Bet v 1 induced significant IL-5 and IL-13 expression, albeit significantly lower than alum-adsorbed Bet v 1. In addition, ELP/ELP-Bet v 1 induced significant IL-10 expression but failed to induce significant IL-4, IFN-γ and IL-17A expression.

Next, we performed a placebo-controlled immunogenicity experiment to confirm the ELP/ELP-Bet v 1 results of the pilot experiment and compared it to alum-adsorbed Bet v 1 (**Figure 7A**). Again, the ELP/ELP-Bet v 1 treated mice showed significant IgG_1_ and IgG_2a_ induction 6 days after the first booster injection (**Figures 7C, D**). Alum-adsorbed Bet v 1 induced only significant IgG_1_ levels at the endpoint, comparable to those of the ELP/ELP-Bet v 1 group. At day 13, alum-adsorbed Bet v 1 also induced significant IgG_2a_ levels but these were significantly lower (18.6-fold) than the titers elicited by ELP/ELP-Bet v 1. Noticeably, IgG_1_ levels in the ELP group were slightly but significantly higher than those of the control group at days 13 and 20. However, these levels were comparable at the endpoint and within the variation of background levels of negative control groups from other immunogenicity experiments. Furthermore, ELP/ELP-Bet v 1 did not induce IgE, whereas the alum-adsorbed Bet v 1 treated mice showed significant IgE induction (**Figure 7B**).

**Figure 8:**
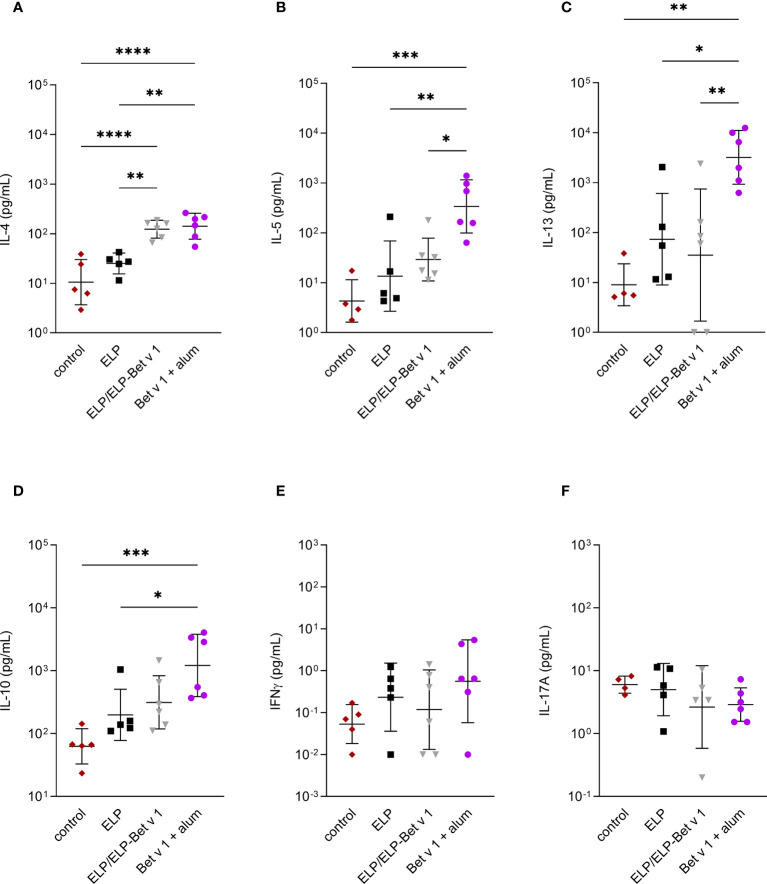
Cytokine expression in lymph node cultures. Expression of IL-4 **(A)**, IL-5 **(B)**, IL-13 **(C)**, IL-10 **(D)**, IFN-γ **(E)** and IL17A **(F)** in *ex vivo* re-stimulated lung draining lymph nodes. *p<0.05, **p<0.01, ***p<0.001 and ****p<0.0001. Displayed are means ± SD.

For alum-adsorbed Bet v 1, the cytokine data revealed a similar pattern compared to the pilot experiment (**Figure 8**). Alum-adsorbed Bet v 1 induced significant expression of IL-4, IL-5, IL-13 and IL-10 but not IFN-γ and IL-17A. In contrast to the pilot experiment, ELP/ELP-Bet v 1 induced significant IL-4 expression which was comparable to that induced by alum-adsorbed Bet v 1. Although the mean expression levels of IL-5, IL-13 and IL-10 were higher than the control group, they were not significantly different.

## Discussion

In this study we investigated ELP/ELP-Bet v 1 micelles as a possible candidate to replace alum in SCIT. As model allergen we used recombinant Bet v 1, the major birch pollen allergen and one of the most studied allergens in molecular allergology (32). The ELPs were chosen as nanoparticle-based delivery system because the controlled self-assembly allows the design of a well-defined vaccine with regard to active components and physicochemical properties. Allergen-bearing nanoparticles are taken up more efficiently by dendritic cells (DCs) than soluble allergens (33). To increase the uptake of the vaccine further, we designed small particles (<200 nm) which are known to be transported to the lymph nodes and taken up by lymph node resident DCs (34). These nanoparticles may also induce an inflammatory response in the injection site vicinity, inducing a cascade of immune reactions and ultimately increasing the allergen-specific immune response (11).

We successfully produced spherically shaped ELP/ELP-Bet v 1 micelles of a 45 nm size that met our predefined criteria. Furthermore, characterization of these particles showed a 24°C CMT which is below body temperature of both mice and humans, promoting micelle formation inside the body. This *in vivo* micellization is further promoted by the CMC, which is 465-fold lower than the concentration used for the immunogenicity study. Moreover, our data shows that micelles spontaneously form when a cold concentrated solution is diluted into 37°C Tyrode’s buffer. The humoral immune response differences in the pilot immunogenicity experiment between ELP-Bet v 1 and ELP/ELP-Bet v 1 was probably caused by the unfavorable CMC and CMT of ELP-Bet v 1 which hampered *in vivo* generation of nanoparticles.

IgE-mediated side effects are an important safety issue of SCIT. Current SCIT vaccines on the market are often made hypoallergenic by modifying IgE epitopes *via* chemical modification, so-called allergoids (35). However, these efforts of making SCIT vaccines hypoallergenic might also reduce the humoral immunogenicity of the vaccine. For instance, IgG antibodies raised against modified allergens might be less effective in blocking IgE binding to natural allergens than IgG antibodies raised against native allergens. The maximum IgE inhibition achieved by our ELP/ELP-Bet v 1 micelles in the ImmunoCAP inhibition assay was comparable to free Bet v 1 which indicates that many, if not all, of the IgE epitopes were still preserved. Nevertheless, their IgE cross-linking capacity was altered, as shown by the RBL β-hexosaminidase release assay. ELP/ELP-Bet v 1 was both hyper- and hypoallergenic, depending on the tested antigen concentrations. Possibly, below the CMC ELP conjugation to Bet v 1 results in more difficult IgE crosslinking due to steric hindrance, while above the CMC repetitive display of Bet v 1 facilitates IgE crosslinking, suggesting a higher risk for the development of adverse effects. However, in contrast to Bet v 1 and alum-adsorbed Bet v 1 treated mice, *in vivo *our ELP/ELP-Bet v 1 micelles showed a low frequency of temperature drops after injection in Bet v 1 sensitized mice, comparable to the placebo group (**Figure S12**). A possible explanation is that, compared to a relatively static *in vitro* model, in a more dynamic *in vivo* environment the ELP/ELP-Bet v 1 micelles will be gradually diluted to below the CMC, resulting in disassembled but hypoallergenic ELP-Bet v 1. Another explanation could be that the ELP/ELP-Bet v 1 micelles are already being taken up by other cells at the injection site, e.g. tissue resident macrophages or dendritic cells, before they are able to interact with IgE loaded basophils or mast cells. In short, whether the increased mediator release above the CMC is relevant for the *in vivo* situation remains unclear and more studies are required to measure allergy-associated adverse effects following ELP/ELP- Bet v 1 administration.

Our *in vivo* data confirmed that alum is a typical Th2 adjuvant in mice. In contrast, the ELP/ELP-Bet v 1 micelles showed a weak, Th2-skewed immune response, indicated by variable IL-4 and low IL-5 expression. Moreover, other pro-inflammatory cytokines such as IFN-γ and IL-17A were not induced, suggestive of an overall weak T-cell response. Interestingly, IFN-γ is associated with IgG_2a_ induction but in the alum-adsorbed Bet v 1 and ELP/ELP-Bet v 1 groups IFN-γ was not detected, despite measuring significant IgG_2a_ levels. A possible explanation is that IFN-γ producing T-cells necessary for IgG_2a_ isotype switching were short-lived and therefore not detectable at the study endpoint. In addition, the overall weak pro-inflammatory T-cell response could be related to the non-immunogenic character of ELP that was used in our design and because Bet v 1 requires pollen derived factors to stimulate DCs and induce T-cell polarization (36). Besides a generally weak pro-inflammatory immune response, the ELP/ELP-Bet v 1 micelles also induced a generally weak IL-10 response that was slightly higher compared to placebo treated mice but much lower compared to alum-adsorbed Bet v 1 treated mice. This could be linked to the much weaker pro-inflammatory response of ELP/ELP-Bet v 1, because Th2 cells can also produce IL-10 (37).

In addition to a weak Th2-provoking immune response, the ELP/ELP-Bet v 1 micelles induced early and strong IgG_1_ and IgG_2a_, compared to alum-adsorbed Bet v 1. Noticeably, alum-adsorbed Bet v 1 induced inconsistent humoral responses, which were also observed in other immunogenicity experiments using the same immunization protocol (data not shown). In all experiments, induction of IgGs was only detected after the immunizations and BP challenges. The protocol for our immunogenicity experiments was derived from our sensitization protocol used for our birch pollen allergy SCIT model (2). The difference between the two protocols is that the mice were immunized *via* the subcutaneous route instead of the intraperitoneal route. Immunization *via* the intraperitoneal route typically induces stronger humoral responses than the subcutaneous route. However, in this study the subcutaneous route was chosen to mimic SCIT administrations. Despite using genetically almost identical mice, we occasionally have observed differences between mouse experiments. This could be at least partly caused by inter-experimental variability. Moreover, when using this protocol for another study, albeit with a lower Bet v 1 dose, alum-adsorbed Bet v 1 did not induce strong antibody levels but the SCIT candidate showed an early and strong humoral response. Our results are in line with other murine studies that used alum-adsorbed Bet v 1 administered *via* the subcutaneous route. These protocols were longer but showed a relatively slow and variable induction of IgG antibodies which were mostly observed after the immunizations and not in between (38, 39). Using an extended protocol could improve the detection of alum-adsorbed Bet v 1 induced antibodies. Nevertheless, it is expected that using an extended protocol our ELP/ELP-Bet v 1 micelles will induce earlier and stronger humoral responses compared to alum-adsorbed Bet v 1 (39).

The ELP/ELP-Bet v 1 formulation was tested for contaminations to confirm that the increased allergen-specific IgG_1_ and IgG_2a_ responses in the ELP/ELP-Bet v 1 group are caused by the intrinsic adjuvant property of the nanoparticles. The ELP/ELP-Bet v 1 mixture contained minimal endotoxins (**Figure S1**) and a low level of murine Bet v 1-specific IgG originating from the immuno-affinity column. To test whether this would affect the immune response in mice we spiked alum-adsorbed Bet v 1 with the contaminating amount of murine IgG and compared its immune response to non-spiked alum-adsorbed Bet v 1. We did not observe significant differences between the groups (data not shown).

Extrapolation of murine data to the human situation is often challenging. During SCIT in humans, IL-10 produced by regulatory T- and B-cells is able to suppress the ongoing, Th2 associated allergic inflammation (3, 6). In mice, IL-10 was initially associated with Th2 cells but now it has been shown that other cell types are also capable of IL-10 production, including Th1, Th17, CD8^+^ T-cells, natural killer cells, DC, macrophages and mast cells (40, 41). Therefore, an essential next step is to fill the gap between animal models and clinical studies by exploring if our ELP/ELP-Bet v 1 micelles are capable of inducing similar regulatory T-cell responses as observed in humans during SCIT. This can be tested *ex vivo* using human monocyte derived DCs stimulated with ELP/ELP-Bet v 1 micelles that are subsequently co-cultured with T-cells to study T-cell differentiation patterns.

B-cell responses in mice are also different from those in humans. Clinical studies have shown that AIT-induced IgG_4_ antibodies can block IgE binding and subsequent IgE-mediated allergic responses, and is therefore considered a biomarker of AIT efficacy (4, 42–44). Besides blocking IgE, IgG_4_ also prevents IgE-facilitated antigen presentation, has not many effector functions and limited ability to form immune complexes (45). The induction of IgG_4_ antibodies is driven by IL-4 and IL-10 and is associated with the so-called modified Th2 response observed during SCIT (46). Whereas murine IgG_1_ is considered the equivalent of human IgG_4_ (47), both IgG_1_ and IgG_2a_ have shown correlation with decreased allergic symptoms in mice (39, 48). For example, in a birch pollen allergy therapeutic mouse model, it was shown that increasing levels of IgG_2a_, corresponding to a Th1 type immune response, correlated to reduction in airway hyperreactivity (5). Our ELP/ELP-Bet v 1 nanoparticles induced both murine IgG isotypes more strongly than alum-adsorbed Bet v 1. Nevertheless, the following step is to test our ELP/ELP-Bet v 1 micelles in an animal SCIT model data to study their capability of inducing blocking antibodies. In addition, *in vitro* experiments using human B-cells could provide more pre-clinical data regarding the capacity of our ELP/ELP-Bet v 1 micelles to induce favorable human humoral responses associated with successful SCIT.

In summary, the ELPs not only facilitate the manufacturing of a nanoparticle-based vaccine but also induce specific immune responses that may improve the safety and efficacy of SCIT. These findings introduce ELP technology as a promising platform to develop novel, alum-free SCIT vaccines. 

## Data availability statement

The original contributions presented in the study are included in the article/**Supplementary Material**. Further inquiries can be directed to the corresponding author.

## Author contributions

JS, HW, RL, JWS, WJ, RR, and AK conceptualized and designed the study. JS and HW wrote the manuscript. WJ, AK, and RR critically reviewed the draft version of the manuscript. JS synthesized and purified the ELPs and performed the DLS measurements. MM performed the AFM imaging and analysis. MM and GS evaluated the AFM results. AL, HW, and LR performed the animal experiments. AB and LA performed the RBL tests. JAM provided plasmids encoding ELPs, advice regarding ELP purification and characterization, and suggested improvements for analysis. RO developed the ELP-Bet v 1 plasmid. All authors contributed to the article and approved the submitted version.

## Funding

This work was supported by the Nederlandse Organisatie voor Wetenschappelijk Onderzoek (TKI-NCI, grant 731.014.207). The work of the authors has been supported by the Austrian Science Funds (Projects P32189) and by the University of Salzburg priority program “Allergy-Cancer-BioNano Research Centre”. Andrew MacKay was supported by the Gavin Herbert Professorship in Pharmaceutical Sciences at the USC School of Pharmacy.

## Acknowledgments

We thank Prof. Fatima Ferreira for the opportunity to perform the RBL assay at the Division of Allergy and Immunology, Department of Biosciences, Paris Lodron University of Salzburg. JAM was supported by the Gavin Herbert Professorship in Pharmaceutical Sciences at the USC School of Pharmacy.

## Conflict of interest

HW is and JWS was an employee of HAL Allergy BV. JWS is an employee of SeraNovo BV. RR is a consultant for HAL Allergy BV, Citeq BV, Reacta Healthcare Ltd., Angany Inc., Mission MightyMe and AB Enzymes GmbH. RR reports speaker fees from HAL Allergy BV, ThermoFisher Scientific and ALK and has stock options of Angany Inc.

The remaining authors declare that the research was conducted in the absence of any commercial or financial relationships that could be construed as a potential conflict of interest.

## Publisher’s note

All claims expressed in this article are solely those of the authors and do not necessarily represent those of their affiliated organizations, or those of the publisher, the editors and the reviewers. Any product that may be evaluated in this article, or claim that may be made by its manufacturer, is not guaranteed or endorsed by the publisher.
