# Supplementary material for: Bet v 1-displaying elastin-like polypeptide nanoparticles induce a strong humoral and weak CD4+ T-cell response against Bet v 1 in a murine immunogenicity model
Source: Front Immunol. 2022 Oct 6;13:1006776. doi: 10.3389/fimmu.2022.1006776 (PMC9583423; doi:10.3389/fimmu.2022.1006776)
Supplement: Supplementary file 1 [file DataSheet_1.docx]

Supplementary Material

# Supplemental methods

## CD

CD spectra were measured on a JASCO J-815 CD spectrometer connected to a Peltier temperature controller using a quartz cuvette with a path length of 1 mm. The reported spectra are averages of 10 spectra that were measured in quick succession, using a range of 190 to 260 nm with 1 nm intervals and a bandwidth of 1 nm. The protein or polypeptide concentrations were 28.5 µM (Bet v 1), 19.1 µM (ELP) and 24.4 µM (ELP-Bet v 1). The sample was incubated at 25 or 37 °C for 90 seconds prior to starting the measurement. The measured ellipticity values were converted to molar ellipticity (based on the concentration and the number of amino acid residues/molecule).

## Mass spectrometry

The mass of ELP and ELP-Bet v 1 was analyzed by ESI-QTOF on a Nanoacquity UPLC system (Waters) connected to a Synapt G2Si mass spectrometer (Waters). As the stationary phase of the reverse phase LC an Acquity UPLC M-Class 300 µm x 50 mm column was used, packed with BEH C4 material of 1.7 µm diameter and 300 Å pore size particles. After injection of 5 µL sample, a gradient was applied of 10 to 90% acetonitrile (Biosolve) in water, both containing 0.1% formic acid (Sigma Aldrich). Electro-spray ionization (ESI) was used via Nano-spray source with ESI emitters (New Objectives) fused silica tubing 360 µm OD x 25 µm ID tapered to 5 ± 0.5 µm (5 nL/cm void volume). The following settings in positive resolution mode were used: source temperature of 80 °C, capillary voltage 4.5 kV, nano flow gas of 0.25 bar, purge gas 250 L/h, trap gas flow 2.0 mL/min, cone gas 100 L/h, sampling cone 25 V, source offset 25, trap CE 32 V, scan time 3.0 sec, mass range 400-2400 m/z. Lock mass acquiring was done with a mixture of Leu Enk (556.2771) and Glu Fib (785.84265), lockspray voltage 3.5 kV, Glufib fragmentation was used as calibrant. Masslynx software was used for acquisition and Ent3 software for polymer envelope signal deconvolution. The MaxEnt 1 software was used for mass deconvolution of the charge state envelopes.

## LPS content determination

Endotoxin levels were determined by using HEK-blue TLR4 reporter cells (InvivoGen) expressing the human TLR4-MD2 receptor complex (Figure S1). Suspensions of 25 thousand cells in 100 µL were mixed with 100 µL polypeptide or LPS samples at various concentrations in a 96-well plate (Greiner Bio-One). The plate was incubated at 37 °C overnight, resulting in the activation of the TLR4 pathway, which was detected by secretion of embryonic alkaline phosphatase (SEAP). The SEAP levels were measured with a QUANTI-Blue assay. First, 20 µL of the cell supernatants were incubated with 180 µL QUANTI-Blue solution (rep-qb1, InvivoGen) for 2 hours at 37 °C. Next, the optical density (OD) at 650 nm was measured with a Tecan i-control 1.7.1.12 plate reader.

## ELP-Bet v 1 nanoparticles in a murine SCIT model

Female BALB/c mice were sensitized via intraperitoneal injection with 500 µL PBS containing 10 µg Bet v 1 and 1 mg alum using the protocol published by van Rijt et al. 2014. Subsequently, the sensitized mice (n=12, 2 × 6 mice, staggered design) received 8 weekly subcutaneous injections (SCIT) with placebo (PBS + alum: no SCIT), Bet v 1, Bet v 1 + alum, ELP/ELP-Bet v 1 or ELP. Naïve mice were included as control group. The active treatment groups received a dose of 36 µg Bet v 1, equivalent to the amount of Bet v 1 in 300 µg BPE that was chosen as most effective dose for SCIT. Thirty minutes after each SCIT injection the body temperature was measured by rectal probe and compared to the temperature measured before injection.

# Supplementary Figures


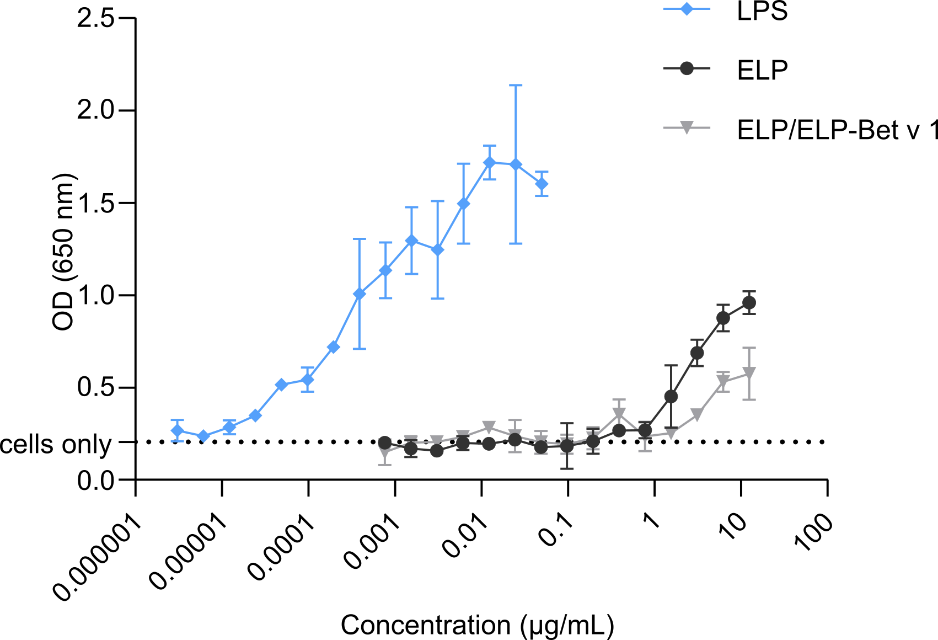


**Figure S1 Endotoxin level determination using mTLR4 HEK reporter cell assay.** Samples were incubated with the cells overnight at 37 °C. Subsequently, SEAP levels were measured with a QUANTI-Blue assay.


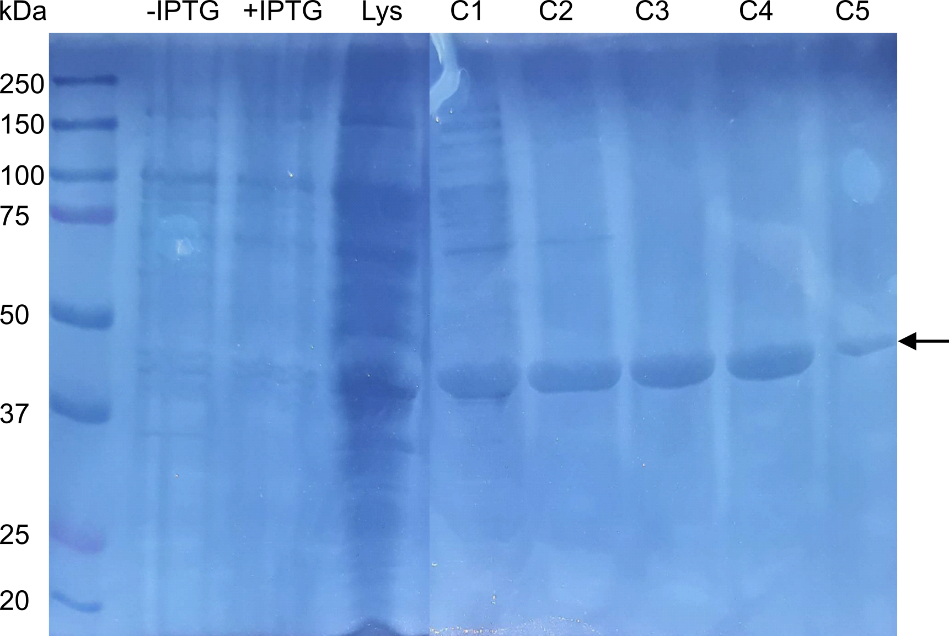


**Figure S2. Expression and purification of ELP.** Samples were analyzed on a 10% SDS-PAGE gel by using CuCl_2_ staining. ELP is clearly visible at about 40 kDa and is indicated with a black arrow. -IPTG is before induction; +IPTG is after induction; C1-5 refer to the samples taken after 1-5 cycles of ITC.


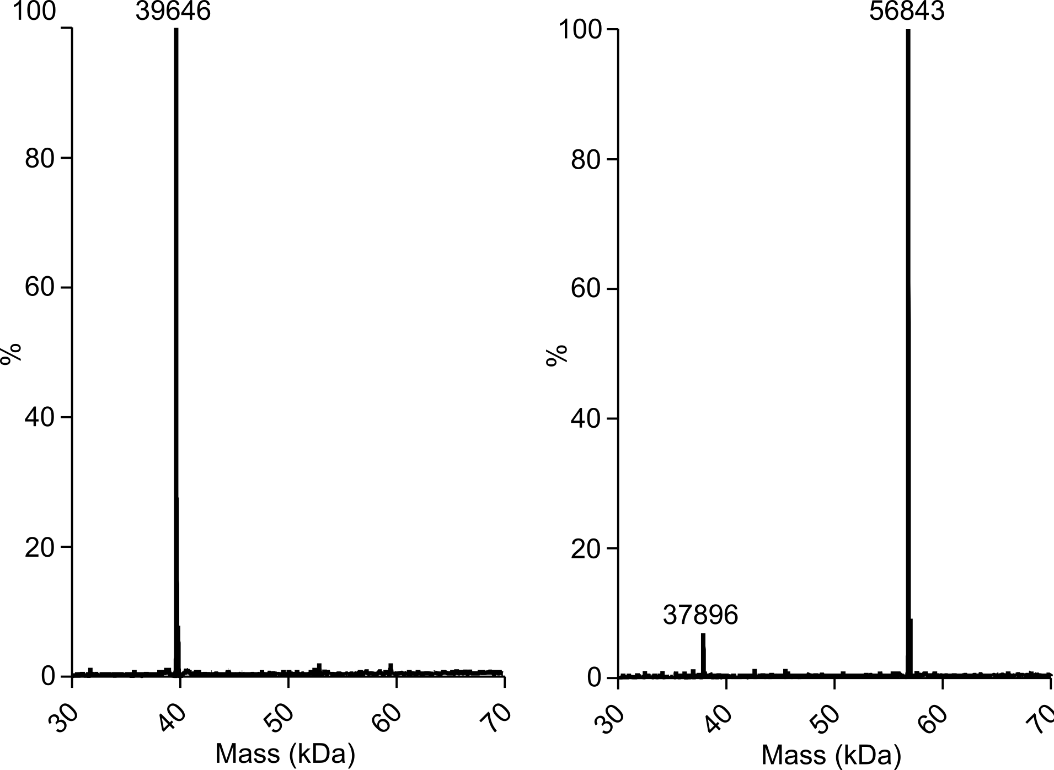


**Figure S3. Mass spectra of ELP (left) and ELP-Bet v 1 (right).** ESI-TOF, measured on an LTQ orbitrap mass spectrometer. Measured masses matched the theoretical masses of the polypeptides without the N-terminal methionine: expected mass ELP is 39642 Da, found 39646 Da; expected mass ELP-Bet v 1 is 56841 Da, found 56842 Da.


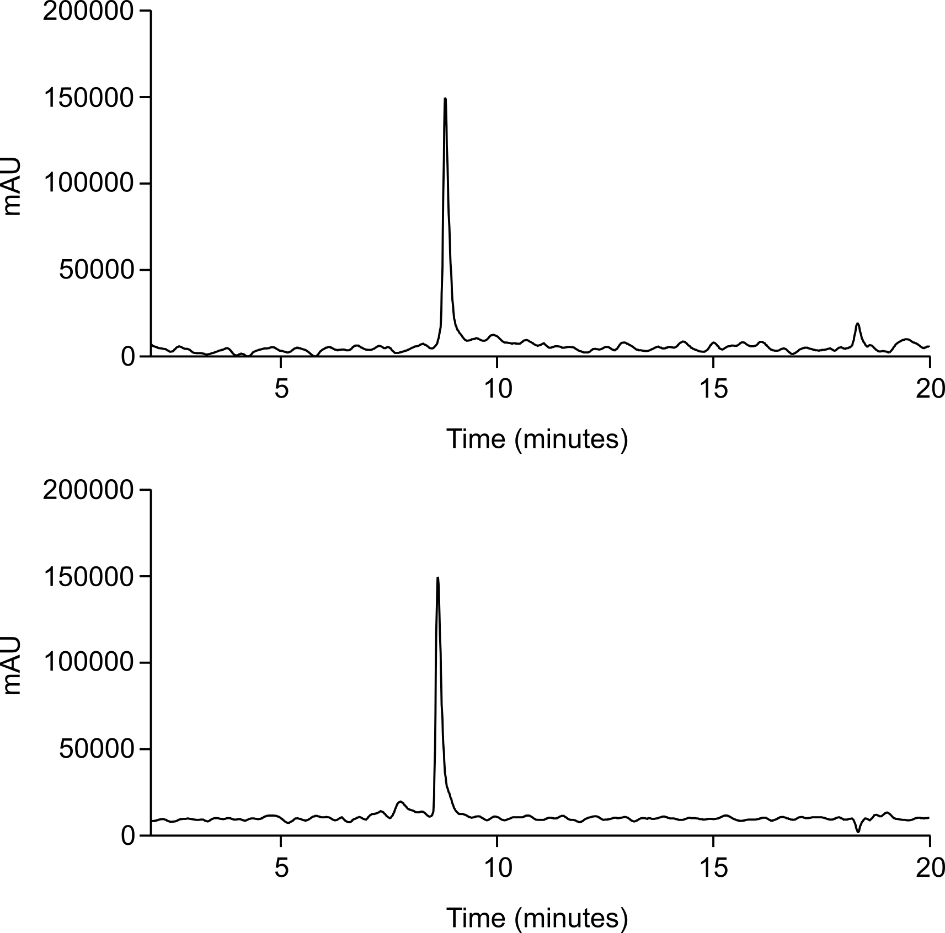


**Figure S4. RP-HPLC analysis of ELP (top) and ELP-Bet v 1 (bottom).** Gradient of 0 to 90% acetonitrile in water containing 0.1% TFA. Trace of ELP-Bet v 1 was normalized to match the peak height of ELP.


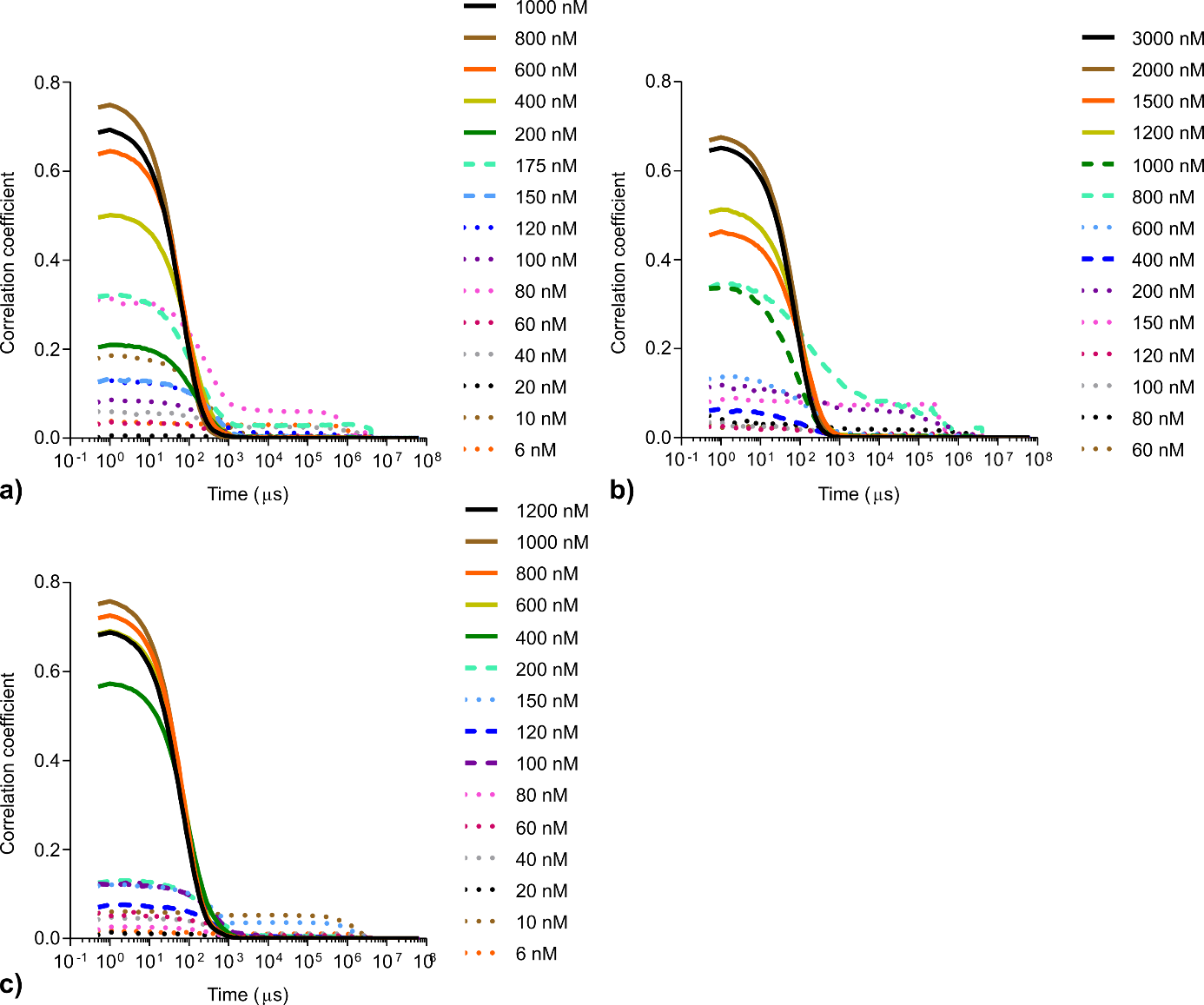


**a**

**Figure S5. Autocorrelation functions of DLS measurements for CMC determination of (a) ELP, (b) ELP-Bet v 1 and (c) ELP/ELP-Bet v 1.** Each graph is an average of three measurements. Solid lines represent samples for which particles were detected; dotted lines represent samples for which particles could not be detected; dashed lines represent samples for which no particles were detected for the first measurement, but particles were detected for the last measurement.

**c**


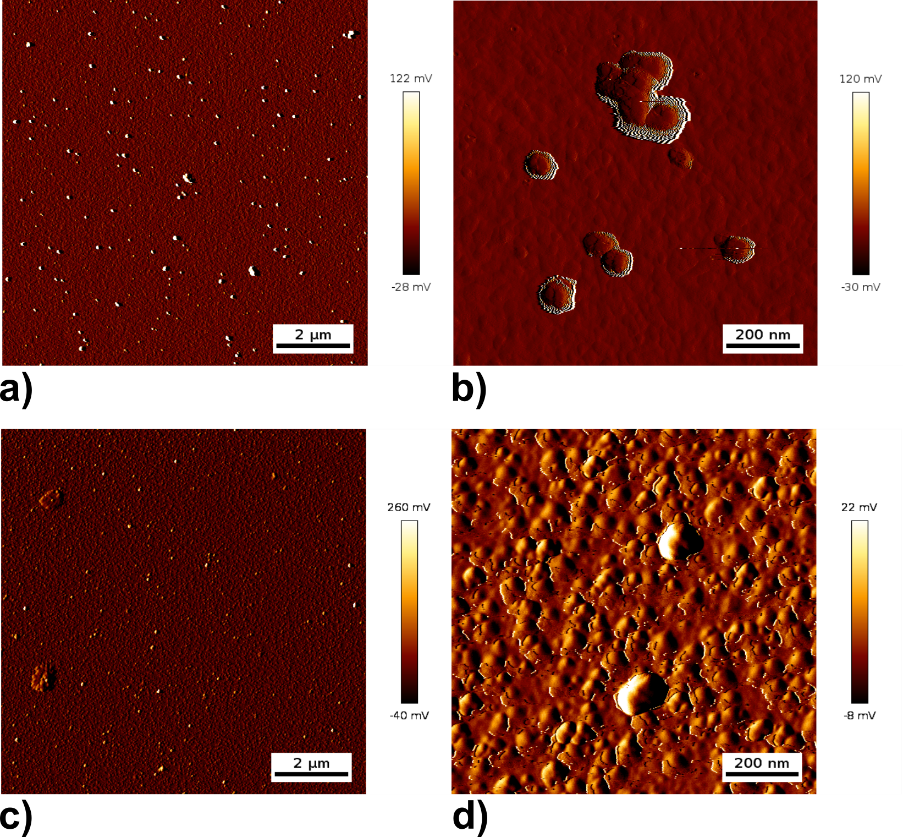


**Figure S6 AFM images and analysis of ELP (a,b) and ELP/ELP-Bet v 1 (c,d).** Polypeptide solutions in water were heated to 37 °C and deposited on a silicon oxide surface. The samples were dried at 37 °C and imaged at medium (a and c) and high (b and d) magnifications in error trace mode.


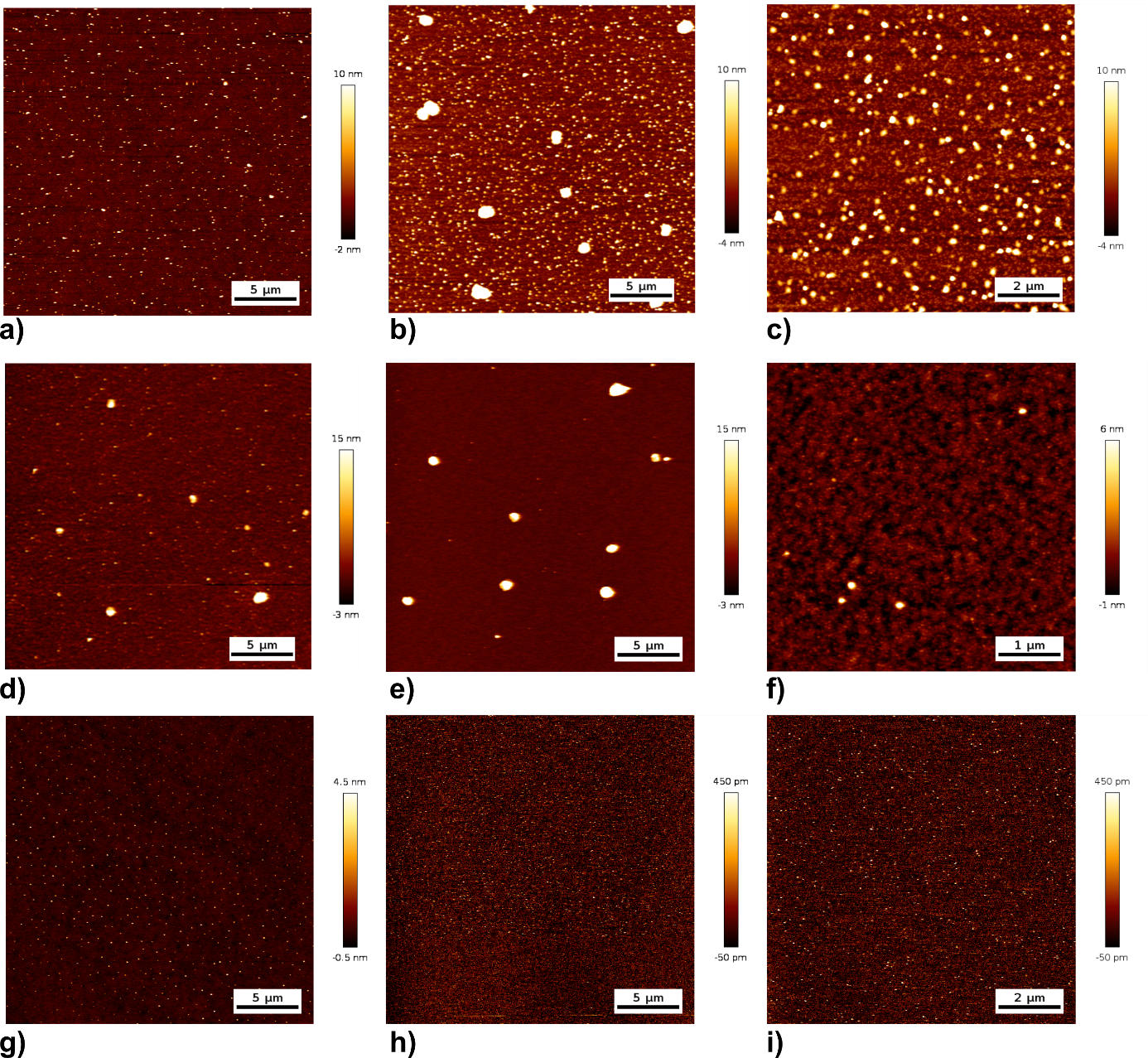


**Figure S7. AFM images of ELP (a-c), ELP/ELP-Bet v 1 (d-f) and a water control (g-i).** Polypeptide solution in water or water control was heated to 37 °C and deposited on a silicon oxide (a, d and g) or mica (b, c, e, f, h and i) surface. The samples were dried at 37 °C and imaged at low (a, b, d, e, g and h) or medium (c, f and i) magnification in height trace mode.


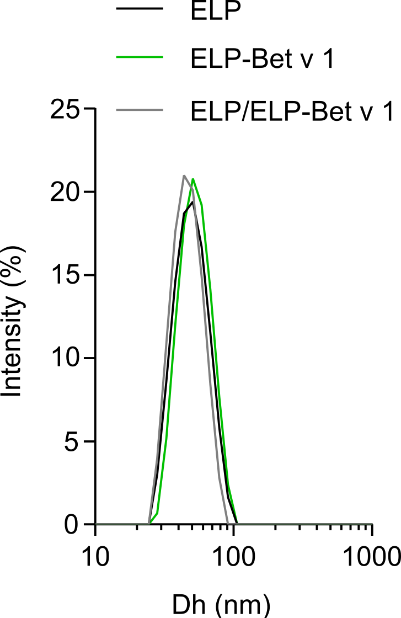


**Figure S8. Size distributions of the Bet v 1-displaying ELP-based micelles.** Polypeptide solutions of 10 µM in phosphate buffered sucrose were measured at 37 °C.


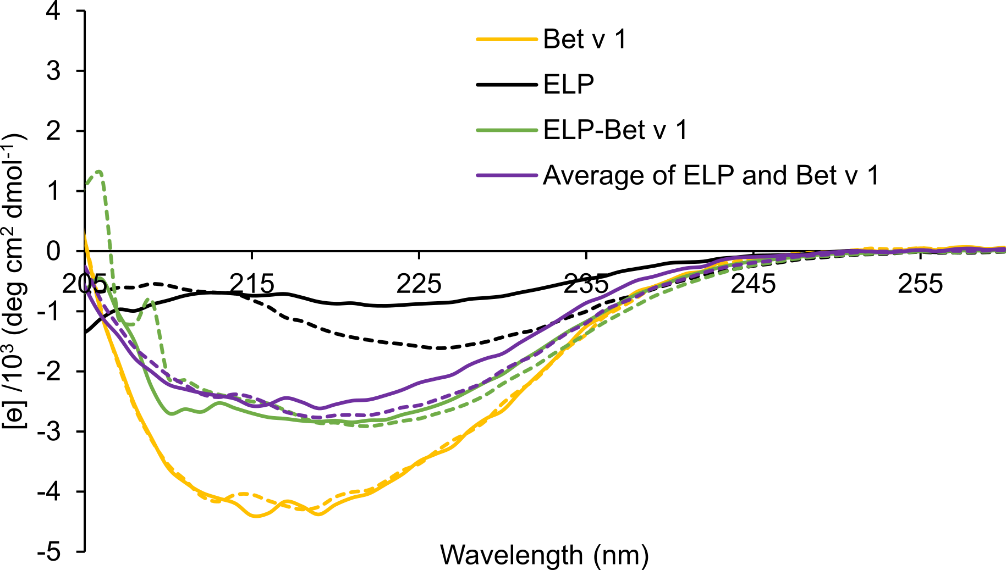


**Figure S9. CD spectra of Bet v 1, ELP and ELP-Bet v 1.** Samples were measured in 10 mM PB at 25 °C (solid lines) and 37 °C (dashed lines); the theoretical average of ELP and Bet v 1 is also shown for both temperatures. The similar curves of the theoretical average and of ELP-Bet v 1 indicate intact of Bet v 1 when conjugated to ELP.

**Figure S10. Serum immunoglobulin levels in immunized mice.** a) Bet v 1 specific IgG_1_ and b) Bet v 1 IgG_2a_ levels at different time points. * p<0.05, ** p<0.01. c) Bet v 1 specific IgE levels at day 31. Displayed are means ± SD. The dotted lines represent average endpoint IgG_1_, IgG_2a_ and IgE levels of a buffer control group from other immunogenicity experiments to indicate background signal.


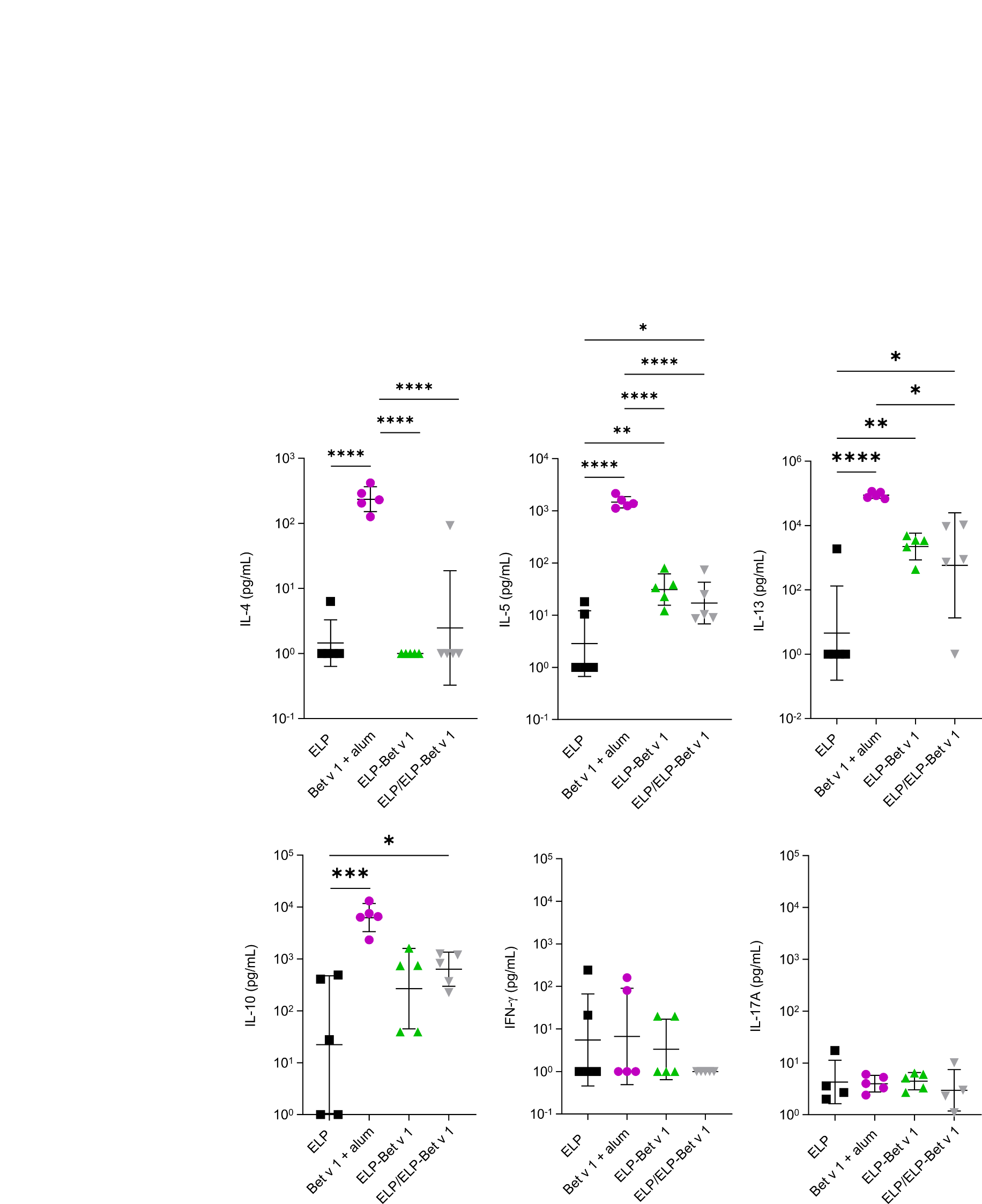


**Figure S11. Cytokine expression in lymph node cultures.** Expression of IL-4 (top left), IL-5 (top middle), IL-13 (top right), IL-10 (bottom left), IFN-γ (bottom middle) and IL17A (bottom right) in *ex* *vivo* re-stimulated lung draining lymph nodes. * p<0.05, ** p<0.01. Displayed are means ± SD.

**Figure S12 Allergenicity of ELP/ELP-Bet v 1 in Bet v 1-sensitized mice.** Female BALB/c mice were sensitized via intraperitoneal injection with alum-adsorbed Bet v 1. Subsequently, the sensitized mice received 8 weekly SCIT administrations with placebo (PBS + alum: no SCIT), Bet v 1, Bet v 1 + alum, ELP/ELP-Bet v 1 or ELP. Naïve mice were included as control group. The cumulative proportion of mice experiencing a drop in body temperature >1°C after each SCIT was greater in the Bet v 1 and Bet v 1 + alum treated groups compared (0.833 after SCIT 8) to the ELP/ELP-Bet v 1 group (0.25 after SCIT 8).
